# KRAB-Zinc Finger Protein ZNF268a Deficiency Attenuates the Virus-Induced Pro-Inflammatory Response by Preventing IKK Complex Assembly

**DOI:** 10.3390/cells8121604

**Published:** 2019-12-10

**Authors:** Yi Liu, Wei Yin, Jingwen Wang, Yucong Lei, Guihong Sun, Wenxin Li, Zan Huang, Mingxiong Guo

**Affiliations:** 1 Hubei Key Laboratory of Cell Homeostasis & State Key Laboratory of Virology, College of Life Sciences, Wuhan University, Wuhan 430072, China; 2 School of Basic Medical Sciences, Wuhan University, Wuhan 430071, China; 3 Hubei Provincial Key Laboratory of Allergy and Immunology, Wuhan 430071, China

**Keywords:** KRAB-ZNF, *ZNF268*, viral infection, IKKα, pro-inflammatory cytokine

## Abstract

Despite progress in understanding how virus-induced, NF-κB-dependent pro-inflammatory cytokines are regulated, there are still factors and mechanisms that remain to be explored. We aimed to uncover the relationship between KRAB-zinc finger protein ZNF268a and NF-κB-mediated cytokine production in response to viral infection. To this end, we established a ZNF268a-knockout cell line using a pair of sgRNAs that simultaneously target exon 3 in the coding sequence of the *ZNF268* gene in HEK293T. HEK293T cells lacking ZNF268a showed less cytokine expression at the transcription and protein levels in response to Sendai virus/vesicular stomatitis virus (SeV/VSV) infection than wild-type cells. Consistent with HEK293T, knock-down of ZNF268a by siRNAs in THP-1 cells significantly dampened the inflammatory response. Mechanistically, ZNF268a facilitated NF-κB activation by targeting IKKα, helping to maintain the IKK signaling complex and thus enabling proper p65 phosphorylation and nuclear translocation. Taken together, our data suggest that ZNF268a plays a positive role in the regulation of virus-induced pro-inflammatory cytokine production. By interacting with IKKα, ZNF268a promotes NF-κB signal transduction upon viral infection by helping to maintain the association between IKK complex subunits.

## 1. Introduction

Inflammation is a pathophysiological immune response that is critical for protecting the host from pathogenic infection [1]. To combat viral infection, cells use various pathogen pattern recognition receptors, such as retinoic acid-inducible gene I-like receptors (RLRs), toll-like receptors, and cyclic GMP-AMP synthase, to detect the nucleic acid of the invading virus [2]. In the case of RNA viruses, such as Sendai virus (SeV) and vesicular stomatitis virus (VSV), once the viral RNA enters the host cell cytosol, RLR family members like RIG-I recognize and bind the viral RNA, activating signaling cascades to induce interferon and pro-inflammatory cytokines that are mainly dependent on the transcription factors IRF3/7 and NF-κB [3]. Less efficient activation of NF-κB may result in insufficient pro-inflammatory cytokine production, leading to chronic infection [4]. For that reason, NF-κB is a central mediator of the inflammatory response that must be fine-tuned during infection.

In the well-established model of NF-κB activation, various stimuli, including viral infection, activate the pro-inflammatory pathway through different upstream receptors and adaptors, leading to the activation of the key regulatory complex: the IκB kinase (IKK) complex. IKK activation results in phosphorylation of the IκB protein, which then undergoes ubiquitination and proteasome-mediated degradation. This leads to the release of cytoplasmic NF-κB dimers, which translocate into the nucleus and initiate target gene (e.g., *TNFα* and *IL-6*) expression [4,5]. Post-translational modifications, such as phosphorylation and ubiquitination, are key to IKK activation [6,7]. Other factors, like HSP-90/Cdc37, which affects the assembly of the IKK complex, also contribute to fully activate the IKK complex [8,9,10]. Although great progress has been made in understanding virus-induced NF-κB activation, other unknown regulators and the underlying mechanisms of efficient IKK activation still need further exploration.

Krüppel-associated box (KRAB) domain zinc finger proteins (KRAB-ZFPs) represent the largest family of transcriptional repressors in humans [11,12]. Characterized by an N-terminal KRAB domain and a C-terminal array of two to 40 C2H2 zinc fingers, KRAB-ZFPs mediate a wide range of biological processes, including embryonic development [13,14], cell differentiation [15], metabolism [16], apoptosis [17,18,19], and cancer [20]. There is growing evidence of a link between KRAB-ZFP and antiviral defense. For example, murine ZFP809 was shown to repress transposable elements originating from murine leukemia virus (MLV), one of the many retroviruses that have invaded and reshaped mammalian genomes during evolution [21]. Both an arms race model and a domestication model have been proposed to explain the relationship between KRAB-ZFPs and their targets [22,23]. Additionally, ZNF10 [24], SZF1, and ZNF557 [25] are reported to inhibit HIV-1 LTR activity and to silence the cancer-causing viruses Epstein–Barr virus (EBV) and Kaposi′s sarcoma-associated herpes virus (KSHV). Those reports provide strong evidence that KRAB-ZFPs play an important role in controlling exogenous and endogenous viruses by targeting viral nucleic acid directly and by modulating repressive chromatin marks on targeted nucleic acid sequences. It is still unknown whether other KRAB-ZFPs regulate antiviral defense in a manner that does not involve direct binding to DNA.

The KRAB-ZFP gene *ZNF268* encodes eight splice variants but mainly produces two protein isoforms: the full-length isoform ZNF268a and the shorter isoform ZNF268b2 [26,27]. Interestingly, *ZNF268* is evolutionarily conserved across primate but lacks homolog in rodent [23], which implies its species-specific functions. Previously, we showed that ZNF268a, which contains a KRAB domain and 24 zinc fingers, acts as a transcriptional repressor [28], while ZNF268b2, which contains the 24 zinc fingers but not the KRAB domain, contributes to cervical carcinogenesis by interacting with IKK, promoting IKKα/β phosphorylation and NF-κB activation [29,30]. ZNF268 has also been implicated in human fetal liver development [31] and hematological malignancy [32,33]. Despite much effort, the function of ZNF268, especially that of ZNF268a, is still poorly defined.

Considering the important role of ZNF268b2 in regulating TNFα-induced activation of NF-κB [29,30], we wondered whether the physiologically relevant ZNF268a would participate in regulating NF-κB activation. In this work, we investigated the function of ZNF268a in the virus-triggered inflammatory response. Using Sendai virus (SeV) and vesicular stomatitis virus (VSV) infection as models, we demonstrated that after infection, ZNF268a binds to IKKα. Instead of increasing the phosphorylation of the two catalytic subunits IKKα and IKKβ, ZNF268a mainly facilitates the assembly of the IKK complex. ZNF268a deficiency leads to insufficient p65 phosphorylation and nuclear translocation. As a result, cells lacking ZNF268a display impaired production of antiviral inflammatory cytokines. Thus, our results reveal ZNF268a as a positive regulator in the virus-activated NF-κB signaling pathway.

## 2. Materials and Methods

### 2.1. Cell Culture, Transfection, and Virus Infection

Human embryonic kidney (293T) cells and human monocytic (THP-1) cells were cultured in Dulbecco′s modified Eagle′s medium (DMEM) and Roswell Park Memorial Institute (RPMI) 1640 medium, both supplemented with 10% fetal bovine serum and 1% (*w*/*v*) each of penicillin and streptomycin. The cultures were grown at 37 °C in a humidified atmosphere containing 5% (*v*/*v*) CO_2_. The day before transfection, cells were seeded into tissue culture plates. DNA transfections of HEK293T cells were performed with Lipofectamine 2000 (Invitrogen, Carlsbad, CA, USA) according to the manufacturer′s instructions. The Lonza Amaxa Nucleofector kit was used to transfect THP-1 cells with siRNAs (30 pmol/sample) using an optimized Nucleofector Program U-001. The siRNA sequences were: siNC, 5′-UUCUCCGAACGUGUCACGUTT-3′; siZNF268a #1, 5′-GGAGUGUGAUGUUGGAGAATT-3′; siZNF268a #2, 5′-GCAGAAGAGUCGCAGAAUATT-3′; siZNF268a #3 5′-GGAAAACTATGTCTTCTTA-3′. To stimulate inflammatory response, cells were infected with Sendai Virus (1 M.O.I.) and VSV (0.01 M.O.I.) for the indicated hours.

### 2.2. CRISPR-Cas9-Mediated Genomic Deletion

We cloned sgRNAs into the pGL3-U6-sgRNA-PGK-puromycin vector [34] and co-transfected the constructs along with pST1374-NLS-flag-linker-Cas9 into HEK293T cells. After allowing 24 h for transfection, we placed the cells under puromycin (1 μg/mL) and blasticidin (3 μg/mL) selection for a minimum of 3 days. We then picked single clones, cultured them, and identified them by PCR analysis. The sgRNA sequences were: sgRNA#1, 5′-CAGCCTATTAAGACGCCATT-3′; sgRNA#2, 5′-TAACTTAACCCTCTGACCTA-3′.

The following primers were used to detect successful genome editing: Primer A, 5′-GTCCCACCTCTCCAAGAACGAAA-3′; Primer B, 5′-CCAGGACTTGGTGATTTTTGGCT-3′; Primer C, 5′-CTGTCAGAGCCTCGTTTGGTTCCTTA-3′; Primer D, 5′-TCAAGAGGCTGAGGCTGGAGGATCACTT-3′. The following primers were used to detect ZNF268a and ZNF268b2 transcripts: E3 Forward, 5′-GTCCCACCTCTCCAAGAACGAAAC-3′; E3 Reverse, 5′-CCAGGACTTGGTGATTTTTGGCTG-3′; E6 Forward, 5′-TTTTGAAGGCTGGAAAGTCCA-3′; E6 Reverse: 5′-CTCCAGAGGATGCAGCAACAA-3′.

### 2.3. Quantitative Real-Time PCR

We extracted total RNA from cells using TRIzol reagent (Thermo Fisher Scientific, Waltham, MA, USA) according to the manufacturer′s instructions. We reverse transcribed the RNAs into cDNA using Hifair II 1st Strand cDNA Synthesis SuperMix for qPCR (gDNA digester plus). Real-time PCR was conducted with SYBR Green (Applied Biosystems, Foster City, CA, USA) on an Applied Biosystems ABI7500 real-time PCR system (Thermo Fisher Scientific, Waltham, MA, USA) following standard protocols. Quantitation of all target gene expression levels was normalized to GAPDH expression. The primer sequences used were as follows: ZNF268a Forward, 5′-CAGCTTCTATTTGGGTCCCAC-3′, ZNF268a Reverse, 5′-ATTCTGCGACTCTTCTGCTTC-3′; TNFA Forward, 5′-CCTCTCTCTAATCAGCCCTCTG-3′; TNFA Reverse, GAGGACCTGGGAGTAGATGAG; IL6 Forward, 5′-ACTCACCTCTTCAGAACGAATTG-3′; IL6 Reverse, 5′-CCATCTTTGGAAGGTTCAGGTTG-3′; GAPDH Forward, 5′-GGAGCGAGATCCCTCCAAAAT-3′; GAPDH Reverse, 5′-GGCTGTTGTCATACTTCTCATGG-3′.

### 2.4. Enzyme-Linked Immunosorbent Assay

The concentrations of TNFα and IL-6 in culture supernatants were measured using ELISA kits (eBioscience and R&D Systems, respectively).

### 2.5. Luciferase Assays

HEK293T cells were seeded into 24-well plates (1 × 10^5^ cells/well). After 24 h, the cells were transfected with the indicated plasmids (400 ng), the NF-κB reporter plasmid (100 ng) and the Renilla luciferase plasmid (5 ng) as control. After transfection for 24 h, the cells were infected with SeV for 16 h and luciferase activity was measured with the Dual-Luciferase Reporter assay kit (Promega Corporation, Madison, WI, USA). We normalized the data by calculating the ratio between firefly luciferase activity and Renilla luciferase activity.

### 2.6. Immunofluorescence and Confocal Microscopy

Cultured cells were fixed with 4% (*w*/*v*) paraformaldehyde for 30 min at 4 °C and permeabilized with 0.5% (*v*/*v*) Triton X-100 containing 1% BSA for 30 min at room temperature. Subsequently, the cells were incubated with primary antibody in buffer containing 1% (*w*/*v*) BSA and 0.05% (*v*/*v*) Triton X-100 overnight, followed by incubation with AlexaFluor592-conjugated secondary antibody (Thermo Fisher Scientific) for 1 h at 37 °C. The cells were then washed three times, and the nuclei were stained with DAPI. Images were acquired using Leica TCS SP8.

### 2.7. Cytoplasmic and Nuclear Protein Extraction

Cells were homogenized in hypotonic buffer (10 mM HEPES [pH 7.9], 1.5 mM MgCl_2_, 10 mM KCl) supplemented with protease inhibitors (MCE) by 20 passages through a 1-mL syringe, followed by centrifugation at 1500× *g* for 5 min at 4 °C. The supernatant contained the cytoplasmic fraction. The pellets were washed three times with hypotonic buffer and lysed with high-salt lysis buffer (20 mM HEPES [pH 7.9], 1.5 mM MgCl_2_, 1.4 M NaCl, 0.2 mM EDTA, 25% glycerol) plus protease inhibitors (MCE). After sonication and centrifugation at 12,000× *g* for 10 min at 4 °C, the supernatant contained the nuclear fraction. The protein concentration of both fractions was measured by BCA, and both fractions were subjected to immunoblot analysis.

### 2.8. Immunoprecipitation and Immunoblot Analysis

Cells were lysed with lysis buffer (20 mM Tris-HCl [pH7.4], 150 mM NaCl, 10% glycerol, 1% NP-40) containing protease inhibitors (MCE) and phosphatase inhibitors (MCE) for 30 min on ice. After centrifugation at 12,000× *g* for 15 min, the protein concentrations of the lysates were measured by BCA assay (Thermo Fisher Scientific). Immunoblot analysis was performed using 10–30 μg samples of the lysates.

For immunoprecipitation, equal amounts of the cell lysates were incubated with Dynabeads Protein G conjugated with specific antibody at 4 °C overnight. The next day, the precipitants were washed four times with lysis buffer, and the immunocomplexes were eluted with sample buffer containing 1× SDS loading buffer for 10 min at 95 °C. The immunoprecipitated proteins were then separated by SDS-PAGE.

The antibodies used for immunoblot analysis, immunoprecipitation, and immunofluorescence were as follows: Anti-DDDDK-tag mAb (Clone: FLA-1), Anti-HA-tag mAb (Clone: TANA2), and Anti-Myc-tag mAb (Clone: My3; all from MBL); HA tag Rabbit Polyclonal antibody (51064-2-AP), p65 RELA Rabbit Polyclonal antibody (10745-1-AP), HSP90 Rabbit Polyclonal antibody (13171-1-AP), CDC37 Rabbit Polyclonal antibody (10218-1-AP), IKBKG Rabbit Polyclonal antibody (18474-1-AP; all from Proteintech); IKKα Antibody (2628), IKKα (3G12) Mouse mAb (11930), IKKβ (D30C6) Rabbit mAb (8943), Phospho-IKKα/β (Ser176/180) (16A6) Rabbit mAb (2697), Phospho-NF-κB p65 (Ser536) (93H1) Rabbit mAb (3033), Phospho-IκBα (Ser32) (14D4) Rabbit mAb (2859), IκBα (L35A5) Mouse mAb (Amino-terminal Antigen) (4814), and NF-κB p65 (D14E12) XP Rabbit mAb (8242; all from Cell Signaling Technology). The affinity-purified antibody of ZNF268a was customized and prepared by Genscript, and the antibody of ZNF268b2 was homemade as described in [27].

### 2.9. Statistical Analysis

Statistical significance between groups was determined by two-tailed Student′s *t*-tests performed using GraphPad Prism 5.0. Differences were considered significant when *p* < 0.05 (* *p* < 0.05, ** *p* < 0.01, *** *p* < 0.001).

## 3. Results

### 3.1. Full-Length ZNF268 Knockout in HEK293T Cells

**Figure 1:**
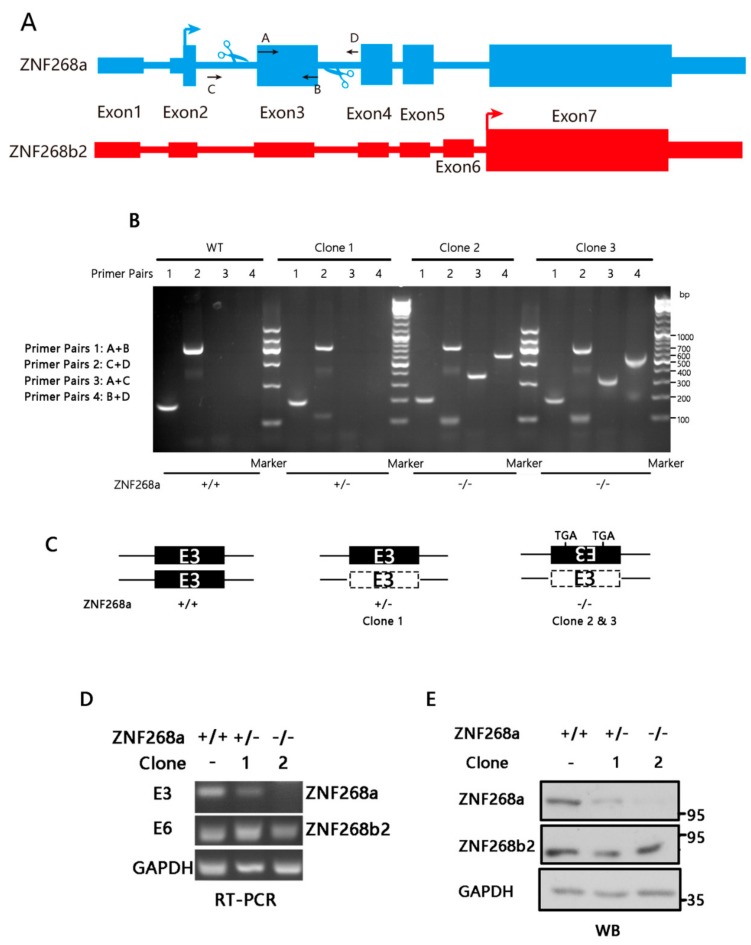
Generation of a ZNF268a-knockout HEK293T cell line. (**A**) Schematic illustration of CRISPR-Cas9-mediated ZNF268a knockout. ZNF268a and ZNF268b2 transcripts are depicted in blue and red, respectively, with the start codon marked by an arrow. Exon 3 of ZNF268a was targeted by two sgRNAs (blue scissors) flanking the 5′ and 3′ ends of the sequence. The targeted exon would be deleted or inverted during non-homologous end-joining repair. (**B**) Genomic PCR products of three individual clones by primer combinations 1: A + B, 2: C + D, 3: A + C, and 4: B + D, analyzed by agarose gel electrophoresis. The 201-bp product of primer pair 1 represented non-deleted exon 3. The ~840-bp product of primer pair 2 also suggested non-deleted exon 3. The small band at ~100 bp indicated that at least one allele was edited. Primer combination 3 and 4 would produce a ~300-bp and ~450-bp bands if an inversion of exon 3 occurred. Clone 1 and 2 were subjected to RT-PCR (**C**) Schematic illustration of the edited alleles of clone 1, 2, and 3. The dotted line box represents the loss of exon 3, the inverted E3 box represents the exon is inverted and 2 in-frame TGA are demonstrated. RT-PCR (**D**) and immunoblot (**E**) using ZNF268 transcript-specific primers and the indicated antibodies, respectively, to confirm the partial or complete knockout of ZNF268a.

Previously, ZNF268b2, the shorter isoform of the encoding gene, was demonstrated to influence TNFα-induced NF-κB activation and to contribute to NF-κB-dependent cervical carcinogenesis [30]. Besides its crucial role in tumorigenesis, NF-κB activation also plays an essential role in virus-induced inflammation. On the basis of that knowledge, we asked whether the longer isoform, or the full-length protein, also plays a role in NF-κB regulation and ultimately orchestrates the inflammatory response to viral infection. To that end, we first used CRISPR-Cas9 to specifically knock out full-length ZNF268. In our initial attempts, we designed an sgRNA targeting the start codon of ZNF268a. That sgRNA appeared to be effective, and we obtained three clones that harbored various small biallelic indels. We wondered, however, whether those single-site frameshifts, which were too close to the 5′ end of the gene, would be efficient enough to induce a loss-of-function phenotype, because it was reported that many frameshift mutations escaped nonsense-mediated decay, and alternative splicing might result in isoforms that compensate or change gene function [35,36]. Therefore, we tried using two sgRNA-mediated genomic deletions instead of just a single disruption. The double-deletion approach had some advantages including rapid PCR identification of edited clones and predictability of loss of function. To make sure that only ZNF268a was knocked out, we designed a pair of sgRNAs to target the flanks of the third exon of the *ZNF268* gene, which is present in the full-length protein but absent in the shorter isoform. By targeting the third exon, we could eliminate ZNF268a and preserve an intact ZNF268b2 (Figure 1A). By analyzing the genomic PCR amplicons of specific primer pairs, we found that the individual clone 1 harbored a single allelic deletion with the other allele unedited, while the individual clones 2 and 3 harbored an exon3-deleted allele and an exon3-inverted allele that produced two in-frame stop codons on the inverted allele, resulting in complete loss of ZNF268a (Figure 1B,C). Indeed, sanger sequencing showed that clone 1 was a heterozygous knock-out clone (+/−), and both clone 2 and 3 were ZNF268a double knock-out (−/−) clones. However, the genomic sequence of clone 2 were identical to that of clone 3, which suggested these two clones might originate from the same parental cell. Therefore, we only used the single knock-out clone 1 (ZNF268a^+/−^) and the double knock-out clone 2 (ZNF268a^−/−^) in the following experiments. The partial or complete loss of ZNF268a in clone 1 and clone 2 were further verified by RT-PCR using primers targeting either exon3 (E3) or exon6 (E6) (Figure 1D). As expected, ZNF268a transcript was largely diminished in clone 1 and was not detected in clone 2, while transcripts encoding ZNF268b2 were similar among the tested samples, indicating our strategy specifically targeted ZNF268a but not affected ZNF268b2. Consistently, Western blot results suggested that ZNF268a was reduced in clone 1 and was lost in clone 2 at the protein level (Figure 1E). Altogether, the results demonstrated the generation of a full-length ZNF268, or ZNF268a-specific, single/double knockout in HEK293T cells.

### 3.2. Full-Length ZNF268 Deficiency Impaired SeV-Induced Proinflammatory Cytokine Production

**Figure 2:**
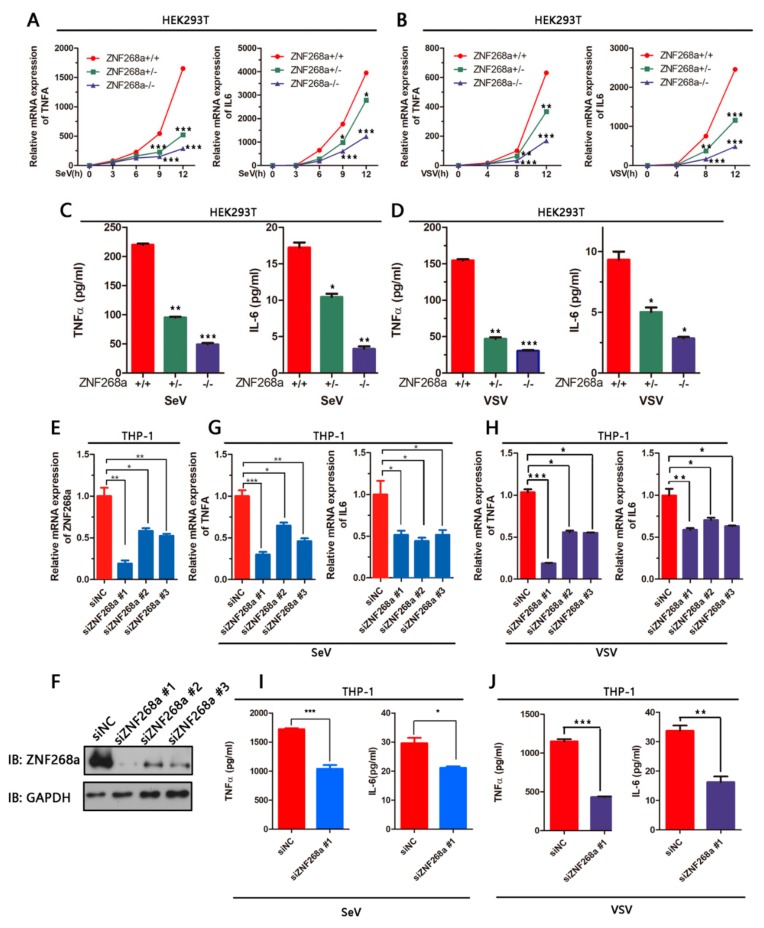
Effects of ZNF268a knockout or knockdown on the virus-induced pro-inflammatory response. Quantification of TNFα and IL-6 mRNA in wild-type, ZNF268a^+/−^ and ZNF268a^−/−^ HEK293T cells in a time-course assay infected by Sendai virus (SeV) (**A**) or vesicular stomatitis virus (VSV) (**B**). (**C**) Quantification by ELISA of secreted proteins in the supernatant after SeV infection for 12 h. (**D**) Quantification by ELISA of secreted proteins in the supernatant after VSV infection for 12 h. (**E**–**H**) THP-1 cells were transfected either by control siNC or by three siRNAs against ZNF268a (siZNF268a #1/#2#3) for 48 h, followed by infection with SeV or VSV for 12 h. The mRNA levels of ZNF268a (**E**), *TNFα* (**G**), and *IL-6* (**H**) were measured by qRT-PCR. (**F**) The silencing efficiency of siRNA was further analyzed by Western blot. (**I**,**J**) *TNFα* and *IL-6* induced by SeV or VSV in THP-1 cells were measured by ELISA, similar to (C) and (D). Data are representative of at least three independent experiments (mean ± standard deviation).

With the establishment of the ZNF268-single/double knockout (ZNF268a^+/−^ or ZNF268a^−/−^) cell lines, we were able to investigate whether full-length ZNF268 is involved virus-induced production of proinflammatory cytokines. First, we infected the ZNF268a^+/−^ and ZNF268a^−/−^ cells with SeV. SeV potently activated NF-κB-dependent expression of TNFα and IL-6 in wild-type HEK293T cells, as measured by qRT-PCR. The expression of those cytokines was strongly impaired in both the ZNF268^+/−^ and the ZNF268^−/−^ HEK293T clones at several time points ranging from 3 h to 12 h after SeV infection (Figure 2A). Results of qPCR experiments using VSV further strengthened the critical role of ZNF268a in regulating those cytokines (Figure 2B). In agreement with the qPCR data, cells lacking ZNF268a could not secrete TNFα and IL-6 properly in response to SeV or VSV infection (Figure 2C,D).

The same was true for TNFα and IL-6 production in THP-1 cells, a monocyte cell line. We designed three different siRNAs against ZNF268a, all of which showed good silencing efficacy at transcriptional level (Figure 2E). There was a significant and accordant reduction of *TNFα* and *IL-6* transcription along with the different down-regulation level of ZNF268a in SeV/VSV- infected THP-1 cells treated with siZNF268a #1, #2 and #3 (Figure 2G,H). We further used to immunoblot analysis to confirm the efficiency of the ZNF268a knockdown in THP-1 cells. We found that consistent with the qPCR data (Figure 2E), all the siRNAs efficiently inhibited ZNF268a protein expression, among which siZNF268a #1 showed the greatest silencing effect (Figure 2F). Therefore, we chose siZNF268a #1 to use in the subsequent ELISA experiments. After cells were transfected with siZNF268#1 via electroporation, they showed substantially less secretion of TNFα and IL-6 into the culture supernatant following SeV or VSV infection (Figure 2I,J). Thus, our experiments using ZNF268^+/−^ and ZNF268a^−/−^ clones confirmed that ZNF268 plays an essential role in SeV-induced pro-inflammatory cytokine expression.

### 3.3. ZNF268a Influenced Cytokine Expression by Regulating the NF-κB Signaling Pathway

**Figure 3:**
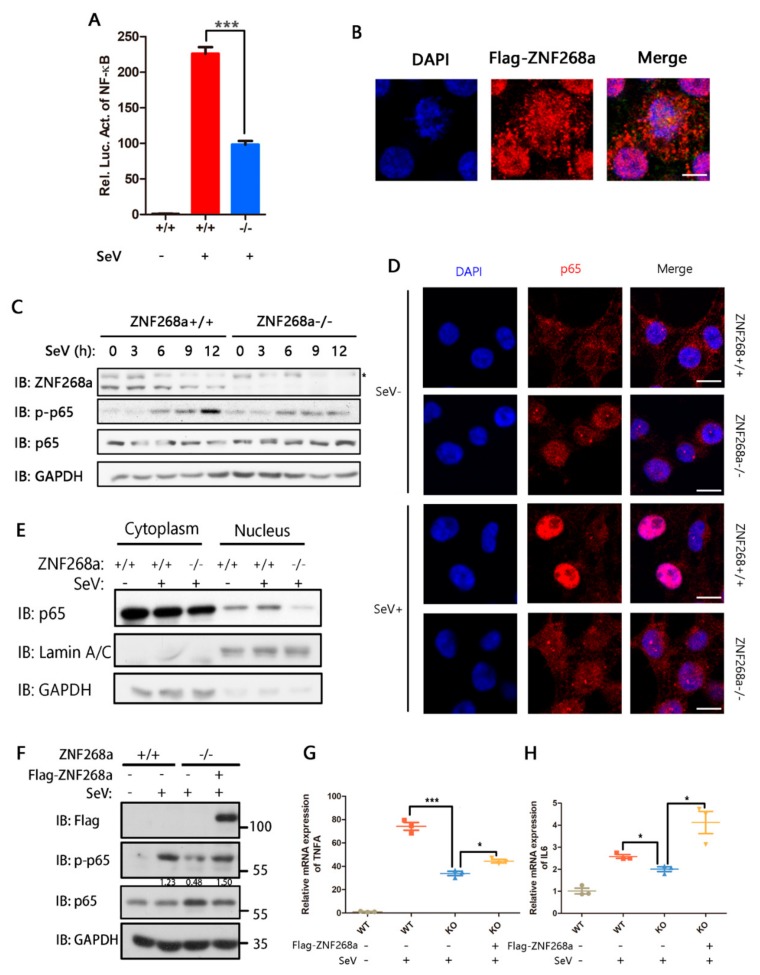
ZNF268a was required for SeV-induced NF-κB activation. (**A**) Wild-type and ZNF268a-knockout cells were transfected with NF-κB luciferase reporter plasmids. Luciferase activity was measured after treatment with SeV for 12 h. (**B**) Representative fluorescent images of ZNF268a-flag-transfected HEK293T cells. Scale bar: 4 μm. (**C**) Western blot analysis of phosphorylated p65 and total p65 in wild-type and ZNF268a-knockout HEK293T cells. An asterisk designates a non-specific band. (**D**) Fluorescent images of p65 in wild-type and ZNF268-knockout HEK293T cells treated with or without SeV for 8 h. (**E**) Treatments similar to those in (**D**) were performed. After 8 h of infection, wild-type and ZNF268a-knockout cells were subjected to subcellular fractionation. Cytoplasmic and nuclear p65 was analyzed by Western blot. (**F**) Flag-ZNF268a construct was transfected into ZNF268a^−/−^ HEK293T cells for 24 h, followed by SeV infection for another 8 h, phosphorylated p65 was detected by Western blot. (**G**,**H**) Treatments similar to those in (**E**) were performed except viral infection period extended to 12 h. The mRNA levels of TNFα and IL-6 were measured by qRT-PCR. Data are representative of three independent experiments (mean ± standard deviation).

After we confirmed the involvement of ZNF268a in the regulation of SeV and VSV -induced, NF-κB-dependent pro-inflammatory cytokines, we next sought to determine the mechanism of the regulation. We used SeV and ZNF268a^−/−^ HEK293T clone as models in our following experiments. We first examined the NF-κB promoter activity by dual-luciferase assay. Upon depletion of ZNF268a, SeV-induced NF-κB promoter activation was severely dampened (Figure 3A). Our group′s earlier work revealed predominant nuclear localization of ZNF268a [37,38]; however, the current immunofluorescence analysis using exogenously flag-tagged ZNF268a revealed a relatively small but clear cytoplasmic distribution of ZNF268a (Figure 3B), which raised the possibility that ZNF268a might regulate NF-κB dependent cytokine production in the cytoplasm by affecting NF-κB signal transduction. Indeed, phosphorylation of NF-κB subunit p65, a hallmark of NF-κB activation, was severely impaired in ZNF268a-knockout cells following viral infection, in contrast to that in their wild-type counterparts (Figure 3C). Consistent with the lower phosphorylation level of p65, ZNF268a deficiency also suppressed nuclear translocation of p65 in response to viral infection (Figure 3D). Besides the evidence provided by observation under confocal microscopy, biochemical subcellular fractionation also suggested that p65 translocation into the nucleus was significantly inhibited in the ZNF268a-depleted cells (Figure 3E). In addition, exogenous ZNF268a reintroduced into ZNF268a^−/−^ cells restored the phosphorylation of p65 upon viral infection (Figure 3F). TNFα and IL6 transcription were also partially or completely rescued in flag-ZNF268a-transfected ZNF268a^−/−^ cells (Figure 3G,H). Collectively, our results indicated that ZNF268a is required for SeV-induced activation of the p65 signaling pathway.

### 3.4. ZNF268a Regulated NF-κB by Associating with IKKα

**Figure 4:**
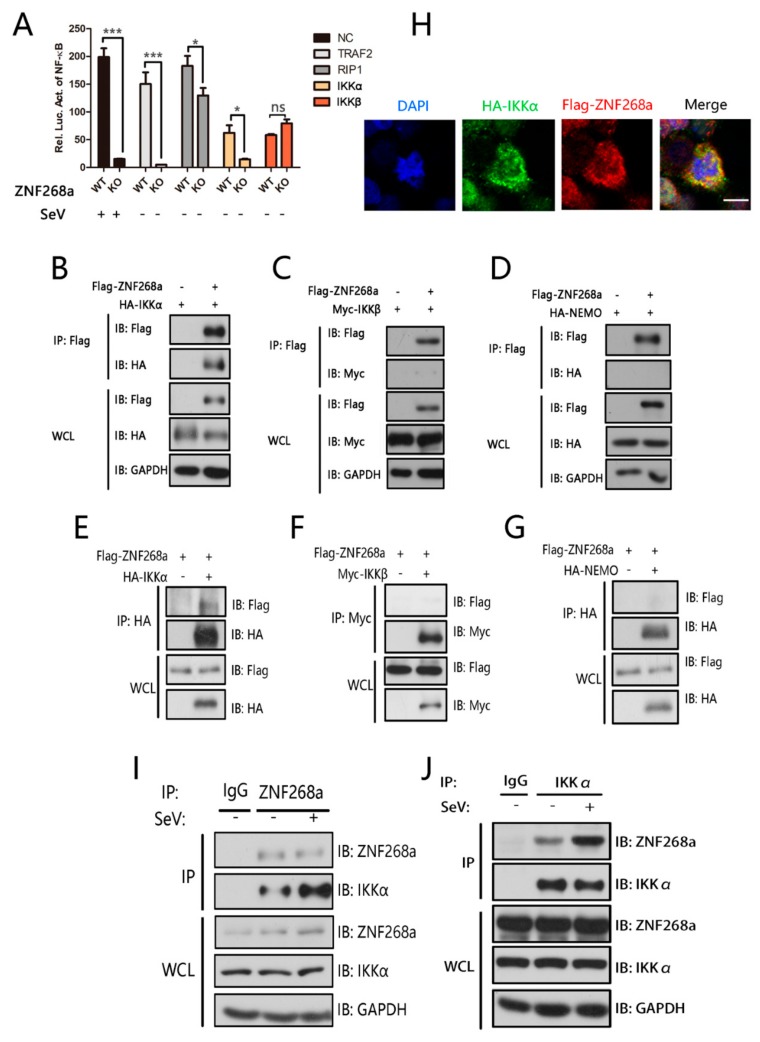
IKKα was targeted by ZNF268a during SeV infection. (**A**) HEK293T cells were transfected with NF-κB reporter plasmid and expression plasmids for TRAF2, RIP1, IKKα, or IKKβ. The luciferase activity was then analyzed. (**B**–**D**) Lysates from HEK293T cells transfected with the indicated plasmids for 24 h were subjected to immunoprecipitation with anti-flag antibody followed by Western blot analysis with anti-HA or Myc antibodies. (**E**–**G**) Immunoprecipitation assay were performed as in (**B**–**D**), except with anti-HA or Myc antibodies during the pull-down and anti-flag in the subsequent western blot. (**H**) Representative fluorescent images of flag-ZNF268a and HA-IKKα inside HEK293T cells. Scale bar: 10 μm. (**I**) With or without SeV infection, HEK293T cells were lysed and immunoprecipitated with anti-endogenous ZNF268a antibody or IgG as negative control, followed by immunoblot with anti-endogenous IKKα antibody. (**J**) Treatments similar to those in (**I**) were performed, except cell lysates were immunoprecipitated with anti-endogenous IKKα or normal control IgG. Data are representative of two or three independent experiments.

Phosphorylation and nuclear translocation of p65 can be induced by enforced expression of a group of several important signal transduction factors, consisting of RIP1, TRAF2, and the IKKα/β/NEMO (also named IKKγ) complex. We asked which of those factors is targeted in the ZNF268a-mediated regulation of p65 activation. We transfected wild-type and ZNF268a-knockout cells with plasmids encoding the signal transduction factors along with NF-κB promoter-driven luciferase reporter constructs. The results suggested that ZNF268a deficiency repressed NF-κB activation induced by RIP1, TRAF2, and IKKα overexpression but not that induced by IKKβ overexpression, indicating that ZNF268a regulates p65 activity by targeting IKKα (Figure 4A).

We next examined the interaction between ZNF268a and the IKK complex subunits by co-immunoprecipitation assay. We transfected HEK293T cells with exogenously flag-tagged ZNF268a (flag-ZNF268a) and HA-tagged IKKα/NEMO (HA-IKKα/NEMO) or Myc-tagged IKKβ (Myc-IKKβ) and performed immunoprecipitation using epitope antibodies. In line with the luciferase assay results, HA-IKKα could be readily detected in immunoprecipitates of flag-ZNF268a, and flag-ZNF268a signal could also be detected in immunoprecipitates of HA-IKKα, suggesting an association between the two proteins (Figure 4B,E). As for IKKβ, the immunoprecipitation assay revealed only very weak interaction, or no interaction, with ZNF268a (Figure 4C,F). NEMO, as an essential adaptor protein in the IKK complex, showed no binding with ZNF268a (Figure 4D,G). We observed co-localization of IKKα and ZNF268a in the cytoplasm by confocal microscopy, which indicated the involvement of cytoplasmic ZNF268a in regulating NF-κB activation (Figure 4H), and, more importantly, further immunoprecipitation experiments using antibody against endogenous ZNF268a revealed enhanced interaction of IKKα and ZNF268a following viral infection (Figure 4I). A similar result was obtained in the reciprocal IP assay using endogenous IKKα antibody (Figure 4J). Taken together, those results clearly suggested that ZNF268a regulates NF-κB activation by physically associating with IKKα in the cytoplasm.

### 3.5. Interacted Domain Mapping of ZNF268a and IKKα

**Figure 5:**
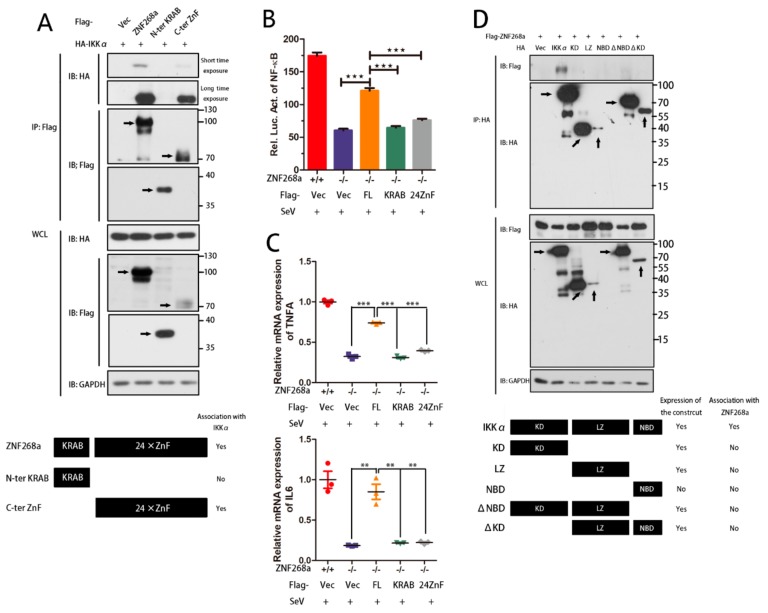
Zinc finger arrays of ZNF268a and the full length IKKα were required for ZNF268a-IKKα association. (**A**) Co-immunoprecipitation and immunoblot analysis of flag-ZNF268a, N-terminal KRAB domain and C-terminal zinc fingers with HA-IKKα. (**B**) Flag-tagged vector, full-length ZNF268a (FL), KRAB domain and 24 Zinc finger arrays were introduced into ZNF268a^−/−^ HEK293T cells. After 36h transfection, the cells were challenged by SeV for 12h before subjecting to dual luciferase assay of NF-κB. (**C**) Similar to (B), except TNFA and IL6 transcripts were measured by RT-qPCR. (**D**) Co-immunoprecipitation and immunoblot analysis of HA-IKKα, Kinase domain (KD), Leucine zinc finger domain (LZ), nemo-binding domain (NBD), deletion of NBD (ΔNBD) and deletion of KD (ΔKD) with flag-tagged ZNF268a. Data are representative of three independent experiments.

We further investigated domains responsible for the interaction between ZNF268a and IKKα. First, we constructed various flag-tagged ZNF268a mutants for the N-terminal KRAB domain and the C-terminal zinc finger arrays. We transfected vectors encoding these mutants into HEK293T cells together with a vector encoding HA-tagged IKKα. Co-IP experiments using flag antibody revealed full length ZNF268a interacted with IKKα as expected, and the zinc finger arrays were strictly required for this interaction as the KRAB domain alone could not bind IKKα. In addition, though zinc finger arrays could bind IKKα, this interaction was not as strong as the full-length protein, suggesting that the KRAB domain also contributed to proper ZNF268a-IKKα interaction (Figure 5A).

Consistent with the Co-IP experiment, being able to bind IKKα, the full-length ZNF268a protein could largely restore the activation of NF-κB. However, with no binding or very weak interaction with IKKα, either the KRAB domain or zinc finger arrays alone was not able to rescue the activation of NF-κB at all in ZNF268^−/−^ cells (Figure 5B). We also measured TNFα and IL-6 mRNA in ZNF268a^−/−^ cells transfected by ZNF268a full-length construct and its variant mutants. The results showed that only the full-length ZNF268a could almost restore these inflammatory cytokines′ expression, and those mutants that failed to bind IKKα were unable to restore the cytokines′ expression (Figure 5C). These results indicated that functionally, an intact ZNF268a was required in the virus-induced activation of NF-κB.

Next, we prepared five mutant HA-tagged IKKα constructs. Using an HA antibody, we performed the Co-IP assay to investigate the interaction of these mutants and Flag-tagged ZNF268a. The result showed that although mutant NBD did not express in our experiments, none of the rest of the four expressed mutants was able to bind flag-tagged ZNF268a, suggesting full IKKα protein was necessary for its interaction with ZNF268a (Figure 5D).

### 3.6. ZNF268a Was Required for Assembly of the IKK Signaling Complex

**Figure 6:**
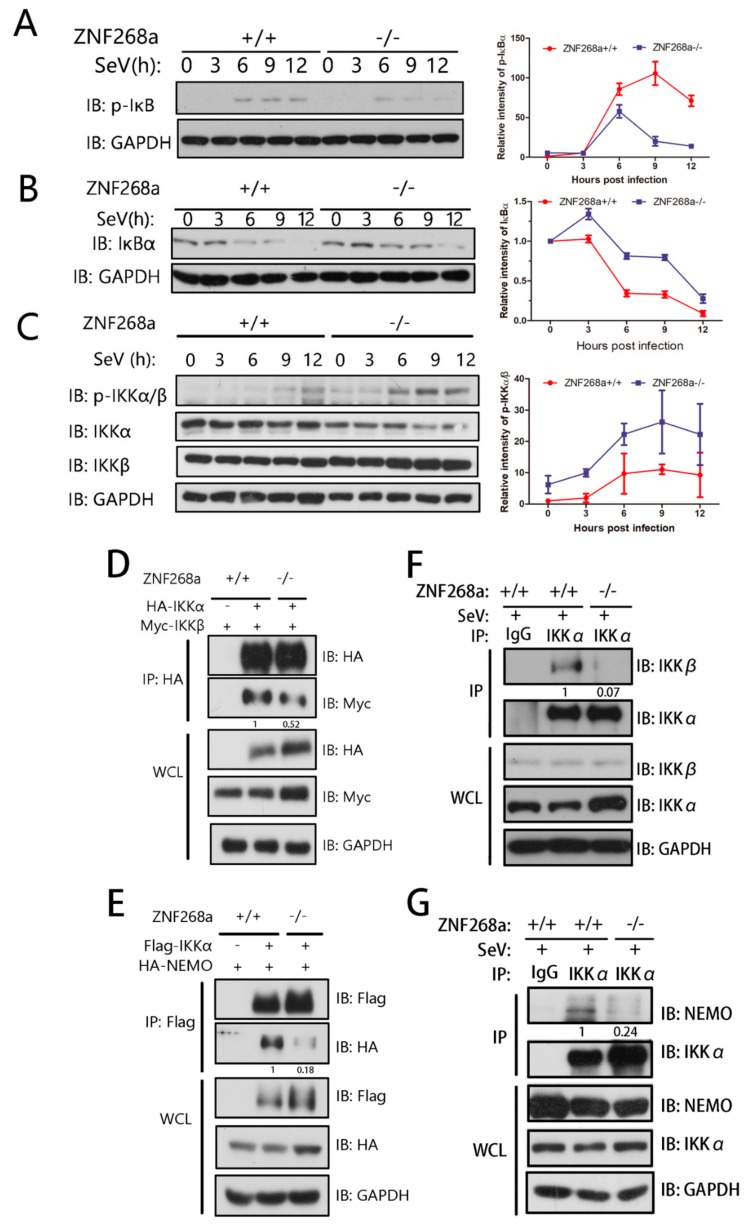
ZNF268a was indispensable for IKK complex assembly, but not required for IKKα/β phosphorylation. (**A**,**B**) Immunoblot analysis of phosphorylated and total IκBα in wild-type and ZNF268a-knockout HEK293T cells infected for the indicated amount of time by SeV. The band intensity of p-IκBα or total IκBα was quantified from two or three independent experiments. (**C**) Immunoblot and band intensity quantification were performed as in (**A**) except phosphorylated IKKα/β was detected. All quantitative data are means ± SEM. (**C**) Co-immunoprecipitation and immunoblot analysis of wild-type and ZNF268a-knockout HEK293T cells co-transfected with flag-IKKα and Myc-IKKβ. Relative gray values of immunoprecipitated Myc-IKKβ/flag-IKKα were measured by ImageJ. (**D**) Endogenous IKKα was immunoprecipitated and endogenous IKKβ in the immunoprecipitates was analyzed by immunoblot, and relative gray values of immunoprecipitated IKKβ/IKKα were quantified by ImageJ. (**E**) Co-immunoprecipitation and immunoblot analysis of wild-type and ZNF268a-knockout HEK293T cells co-transfected with flag-IKKα and HA-NEMO. Relative gray values of immunoprecipitated HA-NEMO/flag-IKKα were measured by ImageJ. (**F**) IP experiment was performed as in (**G**), except that endogenous NEMO was detected and relative gray values of NEMO/IKKα were quantified.

After we demonstrated that ZNF268a interacts with IKKα, we next asked how that interaction affected NF-κB signal transduction. The IKK complex is composed of two kinases, IKKα and IKKβ, with a regulatory subunit, NEMO, all of which are required for the full activation of NF-κB induced by viral infection. At first, we reasoned that ZNF268a might regulate IKKα phosphorylation-dependent NF-κB activation. We found that ZNF268a deficiency led to reduced phosphorylation and delayed degradation of IκBα as expected (Figure 6A,B). However, unexpectedly, the loss of ZNF268a had hardly any inhibitory effect on SeV infection-induced IKKα/β phosphorylation, but instead resulted in a slight or modest increase in the IKKα/β phosphorylation level (Figure 6C). The kinase activities of IKKα and IKKβ depend critically on their phosphorylation [6]. Our result in Figure 6C demonstrated that the activation of IKKs in ZNF268a^−/−^ was comparable to or even slightly higher than that in ZNF268a WT cells. In general, higher IKK activity correlates with stronger activation of NF-κB pathway. However, we observed decreased phosphorylation and slower degradation of IκBα in ZNF268a^−/−^ cells (Figure 6A,B), along with decreased phosphorylation of p65 (Figure 3C), implying impaired activation of NF-κB pathway. These seemingly contradictory results suggested that enzymatic IKK activation alone was required but not sufficient to fully activate downstream signaling components and ZNF268a might regulate IKK by some other mechanism rather than affecting the phosphorylation-dependent kinase activation of IKK.

Besides the catalytic activity of IKKα/β subunits, the formation of IKK complex is another key factor in NF-κB activation. In the canonical NF-κB pathway, IKKα and IKKβ form a heterodimer to phosphorylate their substrate in a NEMO-dependent manner, so we next examined the assembly of the IKK signaling complex in wild-type and ZNF268a-knockout cells. We co-transfected both cells with HA-tagged IKKα and Myc-tagged IKKβ and immunoprecipitated exogenously expressed IKKα to test its association with IKKβ. The results indicated that the interaction was significantly reduced in the ZNF268a-knockout cells (Figure 6D). Similarly, the interaction between exogenous IKKα and NEMO was severely weakened in cells lacking ZNF268a in a flag-IKKα immunoprecipitation assay (Figure 6E). Additionally, IP using endogenous antibody against IKKα clearly suggested dampened interaction in IKK complex (Figure 6F,G). These results suggested the loss of ZNF268a would cause the disruption of the IKK signalosome.

Hsp90 and cdc37 were reported to play important roles in assembly of the IKK Complex [8,9]. We also detected the interaction of ZNF268a and Hsp90/Cdc37. However, we found that endogenous ZNF268a did not bind Hsp90/Cdc37 (Figure S1), suggesting that ZNF268a possibly regulated assembly of the IKK complex independent on Hsp90/Cdc37.

With the results above and the results in Figure 6A,B and C, we concluded that ZNF268a regulates NF-κB signal transduction by facilitating IKK complex assembly, rather than affecting IKK kinase activation.

## 4. Discussion

**Figure 7:**
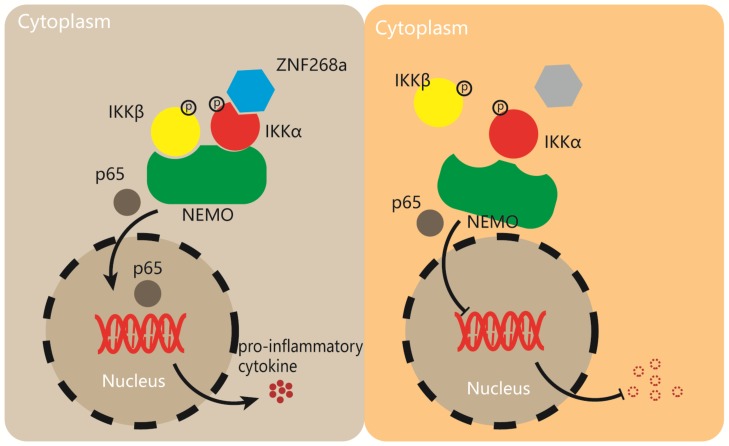
A working model of the regulation of the virus-induced pro-inflammatory response by ZNF268a.

The transcription factor NF-κB plays a critical role in a broad range of physiological and pathological processes, such as inflammation, immune response, cell differentiation, proliferation, and survival. Upon virus infection, a robust inflammatory response is induced via the activation of NF-κB. In this study, we showed that the KRAB-ZFP ZNF268a acts as a specific positive regulator of SeV/VSV-triggered inflammatory responses by promoting the assembly of the IKKα-containing IKK complex. Interactions between IKK subunits were attenuated in the absence of ZNF268a, which suggests that ZNF268a provides an adaptor for the association between IKK proteins. Consequently, ZNF268a-deficient cells produce insufficient pro-inflammatory cytokines in response to viral infection. Our findings reveal a previously unrecognized role of ZNF268a in the regulation of virus-induced inflammation (Figure 7).

As a typical KRAB-ZFP, ZNF268a contains a C-terminal array of 24 zinc fingers and an N-terminal KRAB domain, which is a well-characterized transcription repressive domain. The transcription of *ZNF268* is regulated by cAMP response element-binding protein 2 (CREB-2) [39,40]. The functions of ZNF268a are still a mystery, however. Indeed, a large number of reports have demonstrated that KRAB-ZFPs functions to silence transcription, either by mediation of DNA methylation or by repressive histone modification. In agreement with their roles as transcriptional regulators, KRAB-ZFPs are mainly located in the cell nucleus. Our previous work showed that ZNF268a is mainly located in the cell nucleus [37,38]. Despite that, we found that NF-κB signal transduction, which occurs in the cytoplasm, is inhibited in ZNF268a-deficient cells. Furthermore, although a majority of the ZNF268a proteins are located in the nucleus, we detected a small fraction of ZNF268a in the cytoplasm. Even though it is reported that part of IKKα also resides in the nucleus [41], our confocal microscopy data showed no co-localization of nuclear ZNF268a and nuclear IKKα. On the contrary, we observed the interaction of ZNF268a and IKKα only in the cytoplasm. The fact that ZNF268a lacks a canonical nuclear export sequence (NES) suggests that there is a yet-unknown, NES-independent mechanism underlying the cytoplasmic existence of ZNF268a. In any case, our results indicate a non-canonical function of ZNF268a outside the nucleus.

We demonstrated that IKKα is the main interaction partner of ZNF268a. Previous work focusing on ZNF268b2 [29,30] also identified IKKα/β as interaction partners of that protein. ZNF268a and ZNF268b2 have similar effects on NF-κB activation, which raises the question of whether the two isoforms regulate their targets in a similar way. In view of its amino acid sequence, ZNF268b2 can be considered as the N-terminal KRAB-deleted form of ZNF268a. The overexpression of ZNF268b2 can promote TNFα-induced IKK activation, as it resulted in enhanced phosphorylation of IKKα/β [30]. By contrast, ZNF268a deficiency did not reduce IKKα/β phosphorylation. In fact, we repeatedly found no change or sometimes a slight increase in IKKα/β phosphorylation in ZNF268a KO cells as shown in Figure 6C. These results appeared to be contradictory, but, as a matter of fact, they indicated that the kinase activation of IKKs alone was not sufficient to activate downstream signaling pathway. It was unclear why this occasional slight enhanced phosphorylation of IKKα/β occurred. We speculated that the minor accumulation of phosphorylation on IKKα/β was possibly because the signal transduction in cells was blocked at the IKK level in the absence of ZNF268a. To restore and proceed the transduction, the cells might need a compensatory increase in the phosphorylation of IKKs. Whatever the exact reason was, it was clear that this minor accumulation of phosphorylation of IKKs failed to restore the activation of the pathway in ZNF268a^−/−^ cells, suggesting that besides phosphorylation-dependent activation of IKKs, there must be other key events contributing to activation NF-κB, among which was the assembly of higher order IKK complex. Indeed, in our immunoprecipitation assay (Figure 6D–G), we found that the deficiency of ZNF268a led to a drastic and robust decrease in the IKK subunits association, which implied that ZNF268a affect NF-κB activation via regulating the complex formation rather than via regulating IKK kinase catalytic activity. The KRAB domain may be key to explaining why although both isoforms bind IKK, their underlying regulatory mechanisms differ.

ZNF268b2 is overexpressed in several types of cancer, whereas ZNF268a is not. That implies that the NF-κB activation mediated by ZNF268b2 is probably pathologically relevant, while that mediated by ZNF268a might be more physiologically relevant. The identification of ZNF268a as an important regulator of virus-induced NF-κB activation sheds light on the complicated regulatory processes that modulate inflammation.

Further investigation is needed to determine how ZNF268a is regulated during viral infection, although there is no significant change in ZNF268a transcript expression or total protein expression (data not shown). In addition, even when it is forcibly expressed in cells, the expression efficiency of ZNF268a is quite poor, a feature also seen with ZFP809 [21,42], possibly indicating tight control of ZNF268a within cells, which could explain the reason why sometimes the reintroduction of exogenously expressed ZNF268a only partially restored the activation of NF-κB as seen in Figure 3G and Figure 5B. Detailed knowledge is also needed of whether other ZNF268a-associated proteins play roles in NF-κB regulation, and whether ZNF268a has any effects on the virus-induced interferon-dependent innate immune response by means of crosstalk between the immune-response pathways. Nevertheless, our study provides evidence for a critical role of ZNF268a in pro-inflammatory signaling. In summary, we generated ZNF268a-single/double knockout cell lines with two sgRNA-mediated genomic deletions. The single/double ZNF268a-knockout cells displayed reduced virus-induced production of pro-inflammatory cytokines. THP-1 cells with siRNA-mediated ZNF268a knockdown showed similar results. ZNF268a was found to interact mostly with IKKα. The loss of ZNF268a led to instability of the IKK complex but had little inhibitory effect on IKK phosphorylation. Consequently, the phosphorylation and nuclear translocation of p65 were inhibited, which resulted in impaired production of TNFα and IL-6. ZNF268a therefore appears to be a required factor in the regulation of NF-κB-dependent inflammation.

## Supplementary Materials

The Figure S1 is available online at https://www.mdpi.com/2073-4409/8/12/1604/s1.

## Abbreviations

KRAB-ZFP	Krüppel-associated box domain zinc-finger proteins
NF-κB	nuclear factor binding near the κ light-chain gene in B cells
IκB	Inhibitor of κB
IKK	IκB kinase
SeV	Sendai virus
VSV	vesicular stomatitis virus
CRIPSR-Cas9	clustered regularly interspaced short palindromic repeats-CRIPSR associated protein 9
