# Supplementary figures and images for: KRAB-Zinc Finger Protein ZNF268a Deficiency Attenuates the Virus-Induced Pro-Inflammatory Response by Preventing IKK Complex Assembly
Source: Cells. 2019 Dec 10;8(12):1604. doi: 10.3390/cells8121604 (PMC6953056; doi:10.3390/cells8121604)

**A**

IP: IgG ZNF268a  
SeV: - - +

IP

IB: ZNF268a

IB: Hsp90

IB: Cdc37

IB: ZNF268a

IB: Hsp90

IB: Cdc37

IB: GAPDH

95

95

40

95

95

40

35

WCL

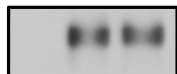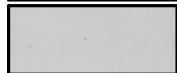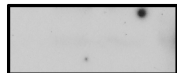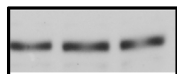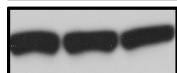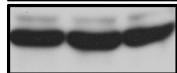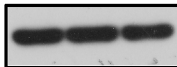

Supplement: Supplementary file 1 [file cells-08-01604-s001.pdf]
